# Cut-Off Values of Hematologic Parameters to Predict the Number of Alpha Genes Deleted in Subjects with Deletional Alpha Thalassemia

**DOI:** 10.3390/ijms18122707

**Published:** 2017-12-13

**Authors:** Diego Velasco-Rodríguez, Carlos Blas, Juan-Manuel Alonso-Domínguez, Gala Vega, Carlos Soto, Aránzazu García-Raso, Pilar Llamas-Sillero

**Affiliations:** 1 Servicio de Hematología, Hospital Universitario, Fundación Jiménez Díaz, IIS-FJD, Universidad Autónoma de Madrid, 28029 Madrid, Spain; cblas@quironsalud.es (C.B.); juan.adominguez@fjd.es (J.-M.A.-D.); gala.vega@quironsalud.es (G.V.); csoto@fjd.es (C.S.); argarciar@quironsalud.es (A.G.-R.); 2 Servicio de Hematología, Hospitales Quirón Públicos, IIS-FJD, Universidad Autónoma de Madrid, 28029 Madrid, Spain; pllamas@fjd.es

**Keywords:** alpha, thalassemia, deletional, cut-off, number of genes, microcytic anemia, differential diagnosis

## Abstract

Most α-thalassemia cases are caused by deletions of the structural α-globin genes. The degree of microcytosis and hypochromia has been correlated with the number of affected α-globin genes, suggesting a promising role of hematologic parameters as predictive diagnostic tools. However, cut-off points for these parameters to discriminate between the different subtypes of α-thalassemia are yet to be clearly defined. Six hematologic parameters (RBC, Hb, MCV, MCH, MCHC and RDW) were evaluated in 129 cases of deletional α-thalassemia (56 heterozygous α^+^ thalassemia, 36 homozygous α^+^ thalassemia, 29 heterozygous α^0^ thalassemia and 8 cases of Hb H disease). A good correlation between the number of deleted alpha genes and MCV (*r* = −0.672, *p* < 0.001), MCH (*r* = −0.788, *p* < 0.001) and RDW (*r* = 0.633, *p* < 0.001) was observed. The presence of an *α^0^* allele should be discarded in individuals with microcytosis without iron deficiency and normal values of Hb A_2_ and Hb F with MCH < 23.40 pg. Furthermore, MCH < 21.90 pg and/or MCV < 70.80 fL are strongly suggestive of the presence of one *α^0^* allele. Finally, an accurate presumptive diagnosis of Hb H disease can be made if both RDW ≥ 20% and MCH < 19 pg are seen.

## 1. Introduction

Alpha-thalassemia is the most prevalent isolated genetic disorder worldwide [1]. Its geographic distribution is highly variable, and the highest prevalence is seen in several regions of China and Southeast Asia [2,3,4,5,6,7], and also in the Mediterranean and Middle Eastern regions [8]. However, migration flows in the last decades have significantly increased its prevalence in the rest of the world and, consequently, the amount of people needing diagnosis and management of this condition is increasing, especially in developed countries [5,8,9].

The *α*-globin gene cluster is located at the short arm of chromosome 16 (16p13.3) and contains 2 functional *α* genes, a *ζ* gene, 3 pseudogenes (Ψα1, Ψα2, Ψζ1) and a *θ*1 gene of undetermined function [10,11]. Normal subjects have 4 α genes (αα/αα), 2 on each chromosome.

The majority of α-thalassemia cases are caused by deletions of the structural α-globin genes, whereas single point mutations or nucleotide insertions (nondeletional α-thalassemia) are much less frequent [1].

There are four types of deletional α-thalassemia, and their severity is correlated with the number of affected α-globin genes [1,11,12,13,14]. Carriers of only one α gene deletion (α^+^ thalassemia) have slightly decreased values of mean corpuscular volume (MCV) and mean corpuscular hemoglobin (MCH), being sometimes overlapped with normal values [1,10,11,12]. Both homozygosis for α^+^ thalassemia (–α/–α) and the heterozygous form of α^0^ thalassemia (––/αα) cause mild microcytic and hypochromic anemia [1,10,11,12]. These milder forms of α-thalassemia can ameliorate the severity of β-thalassemia major and sickle cell disease when they are co-inherited [15,16,17].

Individuals with the loss of three α genes [Hemoglobin (Hb) H disease], characteristically have microcytic hypochromic chronic hemolytic anemia with acute episodes of hemolysis in response to oxidant drugs and infections, splenomegaly and mild jaundice [6,10,12,18]. When the four α-globin genes are deleted (Hb Bart hydrops fetalis syndrome), fetal onset of generalized edema, severe hypochromic anemia, marked hepatosplenomegaly, extramedullary erythropoiesis, and death in the neonatal period are developed [6,12,18].

Since α-thalassemia carriers show normal levels of Hb A_2_ and Hb F, molecular analysis of the α-globin cluster is required for the diagnosis [1]. The polymerase chain reaction (PCR) is the most common method to diagnose deletional α-thalassemia, although other techniques such as Multiplex Ligation dependent Probe Amplification (MLPA) are widely used [19].

Differences in laboratory parameters of red blood cells (RBC) between subtypes of deletional α-thalassemia have been evaluated, and a promising role as predictive diagnostic tools has been suggested [13,14,20]. However, no cut-off points for those parameters have been defined so far.

The aims of this work were to describe the phenotype of RBC based on laboratory parameters of individuals with any form of deletional α-thalassemia, and to evaluate whether the number of deleted alpha genes can be predicted by precise cut-off points of the hematologic parameters.

## 2. Results

The reliability of the results of the complete blood count, Hb A_2_ and Hb F is guaranteed with daily internal quality control (provided by the manufacturer) and external quality assessment every month (Hemqual program, Sociedad Española Hematología y Hemoterapia). Internal quality controls are performed to guarantee that results of the molecular analysis are also reliable.

Of the 129 cases finally included in the study, 57 (44.19%) were males and 72 (55.81%) females and the median age was 35.50 years (3–81).

According to the number of deleted alpha genes, each individual was allocated to one of the following groups:

1. Loss of one α gene or heterozygous α^+^ thalassemia (–α/αα) (*n* = 56) (43.42%). The α^3.7^ haplotype was observed in 55 cases (19 men, 21 women and 15 children), whereas the α^4.2^ haplotype was found only in 1 woman.
2. Loss of two α genes from different chromosomes (–α/–α) (*n* = 36) (27.90%): 34 subjects with –α^3.7^/–α^3.7^ deletions (11 men, 19 women and 4 children), 1 man with –α^4.2^/–α^4.2^ deletions and 1 man was compound heterozygote for the –α^3.7^/–α^4.2^ mutations.
3. Loss of two α genes from the same chromosome or heterozygous α^0^ thalassemia (––/αα) (*n* = 29) (22.48%): This group was comprised of 16 ––^SEA^/αα individuals (4 men, 8 women and 4 children) and 13 ––^FIL^/αα (2 men, 9 women and 2 children). Neither ––^MED^/αα nor –α^20.5^ individuals were identified. The nationalities of our (––^SEA^/αα) cases were: 11 Filipino and 5 Chinese. All of (––^FIL^/αα) cases from our study were Filipino except one Spanish girl whose mother was Filipino.
4. Loss of three α genes or Hb H disease (––/–α) (*n* = 8) (6.20%): 4 subjects presented the ––^SEA^/–α^3.7^ genotype whereas the ––^FIL^/–α^3.7^ genotype was found in 4 cases. All of them were born in the Philippines, except one Spanish girl whose grandfather was Filipino.

Hematological parameters of the 4 groups are summarized in Table 1.

No Hb Bart hydrops fetalis syndrome was diagnosed in the mentioned period of time.

None of the 40 subjects with normal hematological parameters carried any of the evaluated deletions.

All the hematological parameters showed statistically significant differences (*p* < 0.05) between α^+^-thalassemia carriers (–α/αα) and normal controls (αα/αα) (Table 2). The (–α/αα) group presented significantly higher RBC count, lower Hb, lower MCV, lower MCH lower MCHC and higher RDW. MCV, MCH and MCHC stood out as the most efficient parameters to discriminate between these two groups. MCV showed an AUC of 0.975 and the cut-off point of 82.05 provided a sensitivity of 94.7% and a specificity of 94.9%. MCH showed an AUC of 0.982 and the cut-off point of 27.35 provided a sensitivity of 94.70% and a specificity of 92.30%. MCHC showed an AUC of 0.832 and the cut-off point of 33.35 provided a sensitivity of 77.20% and a specificity of 71.80%.

Statistically significant differences (*p* < 0.05) were observed in all the hematological parameters between α^+^-thalassemia carriers and those subjects with 2 alpha genes deleted ((–α/–α) and (––/αα)) (Table 3). Individuals with two alpha genes deleted presented significantly higher RBC count (5.79 × 10^12^/L vs. 5.53 × 10^12^/L, *p* = 0.016), lower Hb (12.63 g/dL vs. 13.7 g/dL, *p* < 0.001), lower MCV (70.20 fL vs. 77.33 fL, *p* < 0.001), lower MCH (21.89 pg vs. 24.83 pg, *p* < 0.001), lower MCHC (31.21 g/dL vs. 32.11 g/dL, *p* < 0.001) and higher RDW (15.04% vs. 14.07%, *p* < 0.001). Only MCV and MCH demonstrated to be efficient enough to discriminate between these subgroups. MCV showed an AUC of 0.879 and a cut-off point of 74.05 provided a sensitivity of 85.70% and a specificity of 78.50%. MCH showed an AUC of 0.905 and the cut-off point of 23.40 provided a sensitivity of 85.70% and a specificity of 78.50%.

Comparison of (–α/–α) and (––/αα) revealed significantly lower MCV (67.34 fL vs. 72.51 fL, *p* < 0.001) and lower MCH (21.02 pg vs. 22.59 pg, *p* < 0.001) in (––/αα) subjects (Table 4). No significant differences were found in RBC, Hb, MCHC and RDW. MCV showed an AUC of 0.815 and the cut-off point of 70.20 provided a sensitivity of 82.80% and a specificity of 77.80%. MCH showed an AUC of 0.810 and the cut-off point of 21.90 provided a sensitivity of 82.80% and a specificity of 64.90%.

Comparison of (–α/αα) and (–α/–α) vs. (––/αα) was also performed in order to identify parameters that could discriminate subjects at risk of having children with Hb H disease or Hb Bart hydrops fetalis syndrome (Table 5). The (––/αα) group presented significantly higher RBC count (5.95 × 10^12^/L vs. 5.58 × 10^12^/L, *p* = 0.004), lower Hb (12.47 g/dL vs. 13.33 g/dL, *p* = 0.006), lower MCV (67.34 fL vs. 75.45 fL, *p* < 0.001), lower MCH (21.02 pg vs. 23.96 pg, *p* < 0.001) and higher RDW (15.41% vs. 14.33%, *p* < 0.001). An AUC ≥ 0.8 was found only in MCV and MCH. MCV showed an AUC of 0.902 and the cut-off point of 70.80 provided a sensitivity of 85.90% and a specificity of 86.20%. MCH showed an AUC of 0.898 and the cut-off point of 21.90 provided a sensitivity of 84.80% and a specificity of 82.80%.

Comparisons of the hematological parameters of the two most frequent forms of α^0^ thalassemia in our sample are summarized in Table 6.

The Hb H disease (––/–α) group presented a significant degree of anemia (9.47 g/dL), more microcytosis (63.72 fL) and hypochromia (16.16 pg), and a marked anisocytosis (22.61%).

ANOVA test showed statistically significant differences (*p* < 0.001) in Hb, MCV, MCH, MCHC and RDW when carriers of the deletion of 1, 2 and 3 genes were analyzed together. Pearson coefficient showed good correlation between the number of deleted alpha genes and each of the following parameters: MCV (*r* = −0.672, *p* <0.001), MCH (*r* = −0.788, *p* < 0.001) and RDW (*r* = 0.633, *p* < 0.001).

## 3. Discussion

Identification of at-risk couples prior to pregnancy by hematologic parameters could prevent the most severe forms of α-thalassemia, especially if they belong to a population at risk for α^0^ thalassemia (––/αα).

When compared to normal controls, (–α/αα) subjects showed mild microcytosis and hypochromia, and MCV (cut-off 82.05 fL) and MCH (cut-off 27.35 pg) proved to be excellent parameters to discriminate between these two subgroups. A known (–α/αα) subject by definition will transmit at least one *HBA* gene to his/her offspring, thus there is no risk of having children with Hb Bart’s disease. However, the importance of identifying (–α/αα) individuals relies on warning them that, if their couple shows microcytosis, a molecular study of α-thalassemia should be performed prior to having children. Our results in (–α/αα) subjects are in accordance with those published by many authors [13,14,20].

Two parameters (MCH and MCV) stood out as the most efficient to predict the deletion of two alpha genes, being their optimal cut-off values 23.40 pg and 74.05 fL respectively. Despite the detection of two deleted alpha genes has no considerable clinical impact (only mild anemia), it allows an adequate genetic counseling to at-risk couples. Overlapping Hb, RBC, MCHC and RDW values were found in (–α/–α) and (––/αα), but the (––/αα) group presented a significantly higher degree of microcytosis and hypochromia. Although both MCV and MCH showed an AUC ≥ 0.8, their optimal cut-offs could not accurately discriminate between both conditions.

The main issue in subjects with suspected α-thalassemia is the identification of individuals carrying an α^0^ allele ((––/αα) or (––/–α)), since they may have children with Hb H disease or Hb Bart’s disease if their couples have α^+^ thalassemia or α^0^ thalassemia respectively. Presumptive diagnosis of Hb H disease (––/–α) is easy to make, since these subjects are usually symptomatic and show a characteristic phenotype. However, identifying (––/αα) individuals remains a challenge in clinical practice. In our study, MCH had the best AUC (0.905) to discriminate (––/αα) from (–α/αα) and (–α/–α), followed by MCV (0.879). According to our results, the presence of one α^0^ allele should be suspected if the MCH < 21.90 pg and/or the MCV < 70.80 fL.

Among (––/αα) cases, no significant differences were observed between the deletions ––^SEA^ and ––^FIL^, and all of the corpuscular indices were almost identical in these two subgroups (Table 6).

As an example of the impact of immigration flows in the increased prevalence of α-thalassemia in our country, all the subjects from our study who had at least one α^0^ allele were Southeast Asian or had ancestries from this geographic area. Although there have been described several forms of α^0^ thalassemia of local ethnicity (––^MA^, ––^CANT^, ––^SPAN^) in Spain [13], most of α^0^ thalassemia cases in our country are ––^SEA^ or ––^FIL^ deletion described in Asian people. We found interesting the absence of individuals with the ––^MED^ in our study, since it has been previously described in Spain [21,22].

As previously described by several authors [6,12,13,14,18,20], we found a more severe degree of anemia in Hb H disease cases, with Hb levels 9–10 g/dL. In addition to this, all our cases showed MCH < 19 pg. Our phenotypic data related to the size and chromia of their erythrocytes are in agreement with previous reports. Finally, a RDW higher than 20% was found in the 8 cases of Hb H disease included in our study. There are no specific data of RDW values in the different types of deletional α-thalassemia in the work of Villegas et al., although a marked increase in RDW of (––/–α) subjects in comparison to other subgroups is mentioned [13]. Akhavan-Niaki et al. did not evaluate RDW in α-thalassemia subjects [14]. However, Ahmad et al. described a marked anisocytosis, with mean RDW values of 26.20 ± 6.7% [20]. High RDW can be explained by the imbalance in the α/β-globin chain ratio produced in the Hb H disease that leads to ineffective erythropoiesis, since unstable free β-globin chain tetramers precipitate in erythroid precursors [12]. Additionally, their elevated reticulocyte count compared to other forms of α-thalassemia [13,20] can also increase the RDW, since reticulocytes are larger than RBC.

Three parameters are strongly affected by the number of alpha genes deleted: MCV, MCH and RDW. The strongest correlation was observed in MCH (*r* = −0.788), followed by MCV (*r* = −0.672) and RDW (*r* = 0.633). The more alpha genes deleted, the lower values of MCH and MCV, whereas RDW showed an opposite trend. These three parameters had consistently shown statistically significant differences and high values of AUC in most of comparisons between subgroups throughout the statistical analysis, especially MCH. Moreover, the stability of MCH during storage of blood samples is higher compared to MCV [23,24]. Based on this consideration, many authors recommend using MCH instead of MCV to screen for thalassemia. Our results are consistent with this recommendation.

To our knowledge, this is the first study that defines cut-off points of several corpuscular indices to discriminate between the different subtypes of deletional α-thalassemia, adding value to an initial diagnostic approach of these conditions.

There might be three possible drawbacks in our study. First of all, the number of cases analyzed is low. However, it has been enough to find significant differences between subgroups. Secondly, non-deletional α-thalassemia cases were not included, although only a minority is due to point mutations. In addition, finally, not all of the deletional forms of α-thalassemia were evaluated. However, the GAP-PCR used in this study detects the most frequent deletional α-thalassemia determinants. Detection of inclusion bodies with supravital stains was not systematically performed.

## 4. Materials and Methods

Over a 5-year period (April 2012–May 2017), all the deletional alpha-thalassemia cases diagnosed in the Fundación Jiménez Díaz University Hospital by molecular analysis were initially included in this retrospective and observational study (*n* = 174). Since hematological parameters can sometimes be normal in α^+^ thalassemia carriers, 40 subjects with normal hematological parameters were also analyzed as negative controls to evaluate the presence of alpha thalassemia even in cases without microcytosis and hypochromia. All patients were de-identified and anonymized prior to analysis. None of the subjects included had received a blood transfusion in the previous 3 months. All samples were collected in K3-EDTA anticoagulant (Vacutainer™, Becton-Dickinson, NJ, USA), and a complete blood count (CBC), an iron panel (serum iron, ferritin, transferrin, and transferrin saturation index (TSI)), high-performance liquid chromatography (HPLC) and molecular analysis were performed in all samples.

Hb A_2_ levels were lower than 3.5% and Hb F levels were lower than 3.4% in all cases, ruling out heterozygous β-thalassemia and heterozygous δβ-thalassemia. Carriers of α-thalassemia and additional hemoglobinopathy were not included in the study. Subjects with ferritin levels < 20 ng/mL and TSI < 18% were considered to have iron deficiency, and were not included in the statistical analysis. Individuals aged ≤2 years were excluded since it is well known that values of both Hb and MCV are lower in children than in adults [25,26,27].

There was a man with the ––^FIL^/–α^X^ genotype, which means he carried, not only the ––^FIL^ deletion in one allele, but also a non-identified deletion in the other allele. In this case, inclusion bodies were identified with brilliant cresyl blue stain. However, since sequencing or further molecular studies could not be performed, the patient was excluded from the analysis.

All data were extracted from the local laboratory information system.

No signed consent was obtained from the patients since all the tests had been performed as part of their clinical work-up. The study followed the ethical principles of the Helsinki Declaration and was previously approved by the ethical committee of our institution (PIC 94/2017_FJD, Comité de Ética de la Investigación de la Fundación Jiménez Díaz, on 25 July 2017).

### 4.1. Analytical Methods

A GAP-PCR assay of the most frequent deletions that cause α-thalassemia (––^SEA^, ––^FIL^, ––^MED^, –α^20.5^, –α^3.7^ and –α^4.2^) was carried out in all 174 patients as previously described [28], with minor modifications. Genomic DNA was extracted from leukocytes using QIAsymphony system (Qiagen GmbH, Hilden, Germany). Extracted genomic DNA was tested for its quality and quantity using Nanodrop 1000 Spectrophotometer (Thermo Fisher Scientific Inc., Wilmington, DE, USA).

The sequences of the PCR primers are listed in Table 7. Since each of the 6 deletions either partially or completely removes the α2 gene [28], its positive amplification was used to indicate heterozygosis when a deletion allele was also present. Each deletion was tested in a different reaction tube, including positive and negative (H_2_O without DNA) controls. The combinations of primers to detect each deletion are summarized in Table 8. Each 50 µL reaction contained 1× PCR buffer containing Tris-Cl, KCl, (NH_4_)_2_SO_4_, pH 8.7; 1.5 mmol/L MgCl_2_, 1 mol/L betaine (SIGMA, St. Louis, MO, USA), 25 pmol of each primer, 0.2 mmol/L of each dNTP, 2 U of HotStar Taq DNA polymerase (Qiagen GmbH, Hilden, Germany) and 250 ng of genomic DNA. Reactions were carried out on a thermal cycler (SimpliAmp™ Thermal Cycler, Thermo Fisher, Singapore), with an initial 15-min denaturation at 95 °C, 35 cycles of 95 °C for 45 s, 60 °C for 1 min, 72 °C for 2 min 30 s, and a final extension at 72 °C for 5 min. Following amplification, 10 µL of product was electrophoresed through a 1% agarose, 1× TBE gel at 80 V for 1 h, stained in ethidium bromide, and visualized on an ultraviolet transilluminator.

The CBC was performed with the Advia 2120 analyzer (Siemens Medical Solutions Diagnostics, Tarrytown, NY, USA). The following parameters of the CBC were assessed in all subjects: absolute RBC count, Hb, MCV, MCH, mean corpuscular hemoglobin concentration (MCHC) and RBC distribution width (RDW). Ferritin, transferrin, and TSI were measured by chemiluminescence immunoassay in the Advia Centaur (Siemens Medical Solutions Diagnostics). Hb A_2_ and Hb F levels were determined by HPLC on the Tosoh G7 analyzer (Horiba, Tokyo, Japan).

### 4.2. Statistical Analysis

The sample was divided into 3 different groups according to gender and age for the statistical analysis: (1) male and female children from 3 to 16 years; (2) females ≥ 16 years; (3) males ≥ 16 years. Cases were also classified according to the number of deleted alpha genes: 1, 2 or 3.

All measurements were expressed as the median ± standard deviation (SD). The Shapiro-Wilk test was used to assess the normality of our dataset in case of less than 30 samples per group in the comparation. An independent sample t-test was used to compare classical hematologic parameters among the different subgroups. In case of less than 30 samples per group and non-normal distribution, non-parametric tests were used (Mann–Whitney). Additionally, non-parametric tests were used when any of the subgroups compared had less than 30 members. *p* values less than 0.05 were considered statistically significant. Receiver operating characteristic (ROC) curves were plotted in all the parameters that showed significant differences and their area under the curve (AUC) used to evaluate their diagnostic performance. An arbitrary value of AUC ≥ 0.8 was used as the cut-off for considering a variable to be efficient enough to discriminate between the different subgroups. For those variables, a cut-off was selected based on its sensitivity and specificity.

One-way ANOVA test was used to compare the median values of each parameter in three groups of subjects according to the number of deleted alpha genes. Kruskal-Wallis test was performed in the case of parameters of samples whose variances were not equal and groups were very different in size.

The Pearson coefficient was estimated to assess the correlation between each of the hematological parameters and the number of alpha genes deleted. An arbitrary value of *r* ≥ 0.6 was considered a good correlation.

SPSS version 19.0 for Windows (SPSS, Chicago, IL, USA) was used for statistical analysis of the data.

## 5. Conclusions

Unequivocal diagnosis of α-thalassemia can only be made with molecular studies, but corpuscular indices provided by hematological counters can be of great utility as predictive markers of the number of alpha genes deleted. According to our results it is mandatory to discard the deletion of at least two alpha genes in individuals with microcytosis without iron deficiency and normal values of Hb A_2_ and Hb F when MCH levels are lower than 23.40 pg. Additionally, the presence of one α^0^ allele should be suspected with MCH < 21.90 pg and/or MCV < 70.80 fL. In this setting, Hb H disease will be the most likely diagnosis if RDW ≥ 20% and/or MCH < 19 pg are seen. Further prospective validation of these cut-off points in cohorts with larger sample size is needed to establish their real utility in daily clinical practice.

## Figures and Tables

**Table 1:** Hematologic parameters of the different subtypes of deletional α-thalassemia. Data represent mean ± SD (standard deviation).

Parameter	Gender and Age	–α/αα *n* = 56	–α/–α *n* = 36	––/αα *n* = 29	––/–α *n* = 8
RBC (×10^12^/L)	Male	6.0 ± 0.42	5.9 ± 0.64	6.6 ± 0.35	6
	Female	5.1 ± 0.49	5.4 ± 0.59	5.6 ± 0.46	5.8 ± 0.43
	Children 3–16 years	5.4 ± 0.33	5.8 ± 0.14	6.1 ± 0.51	6.2 ± 1.41
Hb (g/dL)	Male	15.3 ± 0.94	14.1 ± 1.07	14.3 ± 1.03	9.6
	Female	12.9 ± 0.98	12.3 ± 0.94	11.8 ± 0.73	9.4 ± 0.61
	Children 3–16 years	13.0 ± 0.92	11.5 ± 0.59	12.3 ± 1.12	9.6 ± 0.56
MCV (fL)	Male	79.4 ± 2.75	74.5 ± 3.33	68.1 ± 2.00	66.3
	Female	78.3 ± 3.60	73.2 ± 3.32	68.6 ± 3.52	64.6 ± 5.90
	Children 3–16 years	75.6 ± 3.82	64.4 ± 3.58	64.0 ± 3.87	61.1 ± 9.33
MCH (pg)	Male	25.8 ± 1.68	23.1 ± 1.09	21.5 ± 1.58	16
	Female	25.1 ± 1.74	22.9 ± 1.13	21.0 ± 0.99	17.2 ± 1.18
	Children 3–16 years	24.2 ± 1.07	20.4 ± 0.86	20.1 ± 0.68	15.8 ± 2.61
MCHC (g/L)	Male	32.3 ± 1.60	31.0 ± 0.98	32.2 ± 2.46	24.2
	Female	32.4 ± 1.45	31.2 ± 1.16	30.7 ± 1.19	25.9 ± 0.95
	Children 3–16 years	31.7 ± 1.38	31.2 ± 0.73	31.5 ± 1.00	25.9 ± 0.35
RDW (%)	Male	13.5 ± 0.80	14.97 ± 1.45	15.08± 1.54	23.3
	Female	13.95 ± 1.28	14.60 ± 1.01	15.81 ± 2.14	21.6 ± 1.40
	Children 3–16 years	14.15 ± 0.96	14.76 ± 0.50	14.61 ± 0.79	21.65 ± 0.92

**Table 2:** Comparison of hematologic parameters of heterozygous α^+^ thalassemia (–α/αα) and normal controls without α^+^ thalassemia (αα/αα). Data represent mean ± SD (standard deviation). *p* values less than 0.05 were considered statistically significant.

Parameter	(αα/αα) *n* = 40	(–α/αα) *n* = 56	*p* Value
RBC (×10^12^/L)	5.20 ± 0.46	5.53 ± 0.53	0.003
Hb (g/dL)	14.69 ± 1.32	12.7 ± 1.33	0.001
MCV (fL)	83.97 ± 1.31	77.33 ± 3.75	<0.001
MCH (pg)	28.32 ± 0.91	24.83 ± 1.68	<0.001
MCHC (g/dL)	33.71 ± 0.91	32.11 ± 1.47	<0.001
RDW (%)	13.37 ± 0.95	14.07 ± 1.07	0.002

**Table 3:** Comparison of hematologic parameters in subjects with loss of 1 alpha gene and those with two genes affected. Data represent mean ± SD (standard deviation). *p* values less than 0.05 were considered statistically significant.

Parameter	Loss of 1 Gene (–α/αα) *n* = 56	Loss of 2 Genes (–α/–α), (––/αα) *n* = 65	*p* Value
RBC (×10^12^/L)	5.53 ± 0.53	5.79 ± 0.63	0.019
Hb (g/dL)	13.7 ± 1.41	12.63 ± 1.35	<0.001
MCV (fL)	77.33 ± 3.75	70.20 ± 4.96	<0.001
MCH (pg)	24.83 ± 1.68	21.89 ± 1.52	<0.001
MCHC (g/dL)	32.11 ± 1.47	31.21 ± 1.30	0.001
RDW (%)	14.07 ± 1.07	15.04 ± 1.52	<0.001

**Table 4:** Comparison of hematologic parameters of homozygous α^+^ thalassemia (–α/–α) and heterozygous α^0^ thalassemia (––/αα). Data represent mean ± SD (standard deviation). *p* values less than 0.05 were considered statistically significant.

Parameter	(–α/–α) *n* = 36	(––/αα) *n* = 29	*p* Value
RBC (×10^12^/L)	5.56 ± 0.61	5.95 ± 0.61	0.064
Hb (g/dL)	12.7 ± 1.33	12.5 ± 1.38	0.420
MCV (fL)	72.51 ± 4.56	67.34 ± 3.88	<0.001
MCH (pg)	22.59 ± 1.44	21.02 ± 1.12	<0.001
MCHC (g/dL)	31.17 ± 1.07	31.25 ± 1.55	0.794
RDW (%)	14.74 ± 1.04	15.41 ± 1.92	0.079

**Table 5:** Comparison of hematologic parameters of subjects with and without an α^0^ allele. Data represent mean ± SD (standard deviation). *p* values less than 0.05 were considered statistically significant.

Parameter	(–α/αα), (–α/–α) *n* = 92	(––/αα) *n* = 29	*p* Value
RBC (×10^12^/L)	5.58 ± 0.56	5.95 ± 0.62	0.004
Hb (g/dL)	13.33 ± 1.45	12.47 ± 1.37	0.006
MCV (fL)	75.45 ± 4.70	67.34 ± 3.88	<0.001
MCH (pg)	23.96 ± 1.93	21.02 ± 1.12	<0.001
MCHC (g/dL)	31.74 ± 1.40	31.25 ± 1.55	0.115
RDW (%)	14.33 ± 1.11	15.41 ± 1.92	<0.001

**Table 6:** Hematologic parameters of (––^SEA^/αα) and (––^FIL^/αα). Data represent mean ± SD (standard deviation). *p* values less than 0.05 were considered statistically significant.

Parameter	(––^SEA^/αα) *n* = 16	(––^FIL^/αα) *n* = 13	*p* Value
RBC (×10^12^/L)	5.96 ± 0.58	5.93 ± 0.67	0.918
Hb (g/dL)	12.4 ± 1.33	12.5 ± 1.39	0.694
MCV (fL)	67.06 ± 4.56	67.64 ± 3.14	0.694
MCH (pg)	20.95 ± 1.34	21.09 ± 0.88	0.745
MCHC (g/dL)	31.29 ± 1.97	31.21 ± 0.99	0.894
RDW (%)	15.29 ± 1.75	15.81 ± 2.01	0.097

**Table 7:** Primers for PCR analysis of common α-thalassemia deletions.

Name	Sequence (5′-3′)	Nucleotides (GenBank ID NT_010393)
FIL-F	TGCAAATATGTTTCTCTCATTCTGTG	140821-140846
FIL-R	ATAACCTTTATCTGCCACATGTAGC	172662-172638
20.5-F	GCCCAACATCCGGAGTACATG	147041-147061
3.7/20.5-R	AAAGCACTCTAGGGTCCAGCG	167719-167699
MED-F	TACCCTTTGCAAGCACACGTAC	152260-152281
MED-R	TCAATCTCCGACAGCTCCGAC	170340-170320
SEA-F	CGATCTGGGCTCTGTGTTCTC	155257-155277
SEA-R	AGCCCACGTTGTGTTCATGGC	175909-175889
4.2-F	GGTTTACCCATGTGGTGCCTC	159269-159288
4.2-R	CCCGTTGGATCTTCTCATTTCCC	165142-165120
α2/3.7-F	CCCCTCGCCAAGTCCACCC	161883-161901
α2-R	AGACCAGGAAGGGCCGGTG	163685-163667

**Table 8:** Combinations of primers to detect each deletion.

Fragment	Primers	Size (bp)
FIL deletion	FIL-F + FIL-R	1166
SEA deletion	SEA-F + SEA-R	1349
20.5 deletion	20.5F + 3.7/20.5-R	1007
3.7 deletion	α2-3.7-F + 3.7/20.5-R	2022–2029
4.2 deletion	4.2F + 4.2-R	1628
MED deletion	MED-F + MED-R	807
α2 gene	α2/3.7-F + α2-R	1800
